# Supplementary material for: Interplay between material properties and cellular effects drives distinct pattern of interaction of graphene oxide with cancer and non-cancer cells
Source: J Nanobiotechnology. 2025 May 30;23:393. doi: 10.1186/s12951-025-03400-3 (PMC12123884; doi:10.1186/s12951-025-03400-3)
Supplement: Supplementary file 1 — Supplementary Material 1 [file 12951_2025_3400_MOESM1_ESM.docx]

**Supporting information**

**Table S1:** Summary of the physico-chemical characterisation of s-GO and us-GO.

| **Parameter** | **Technique** | **s-GO** | **us-GO** |
| --- | --- | --- | --- |
| Lateral dimension | Optical microscopy | Non detectable (< 2 μm) | Non detectable (< 2 μm) |
|  | SEM | 25 nm – 2.4 μm  95% < 1.2 μm  Mean = 232 nm  [n = 742] | 10 nm – 503 nm  95% < 250 nm  Mean = 48 nm  [n = 636] |
|  | AFM | 25 nm – 1.3 μm  95% < 625 nm  Mean = 84 nm  [n = 775] | 20 nm – 790 nm  95% < 210 nm  Mean = 59 nm  [n = 4268] |
| Thickness | AFM | 1–2 nm | 1–2 nm |
| Optical properties | Absorbance | ε_230_ (mL μg^− 1^ cm^− 1^) = 0.038 | ε_230_ (mL μg^− 1^ cm^− 1^) = 0.041 |
|  | Fluorescence λ_600_ (λexc_525_) | 1.056*C_GO_ (μg/mL) | 0.915*C_GO_ (μg/mL) |
| Degree of defects (I_D_/I_G_) | Raman | 1.19 ± 0.02 | 1.18 ± 0.03 |
| Peak (2θ)  Interlayer distance (nm) | XRD | 12.81^o^  0.70 | 11.53^o^  0.76 |
| Zeta potential | Electrophoretic mobility | -50.8 ± 1.1 mV | -53.5 ± 2.2 mV |
| Functionalisation degree | TGA | 30–75 ^o^C: 7% (water)  200–250 ^o^C: 24%  250–950 ^o^C: 18%  Total: 42% | 30–75 ^o^C: 7% (water)  200–250 ^o^C: 23%  250–950 ^o^C: 13%  Total: 36% |
| Chemical composition  Purity (%C + %O), C:O ratio, π-π*, *O-C = O*, *C = O*, *C-O*, *C = C* | XPS | C: 70.7%, O: 25.5%, N: 1.8%, S: 1.4%, B: 0.6%  96.2%, 2.8, 0.2%, 4.1%, 3.2%, 46.5%, 46.0% | C: 73.0%, O: 24.8%, N: 0.4%, S: 1.4%, B: 0.4%  97.8%, 2.9, 0.2%, 5.8%, 7.5%, 37.3%, 49.1% |

SEM = scanning electron microscopy, TEM = Transmission electron microscopy, AFM = atomic force microscopy, ε_230_ = molar absorption coefficient at 230 nm, λ_600_ = emission at 600 nm, λexc_525_ = excitation at 525 nm, C_GO_ = graphene oxide concentration, XRD = X-ray diffraction, TGA = thermogravimetric analysis, XPS = X-ray photoelectron spectroscopy.


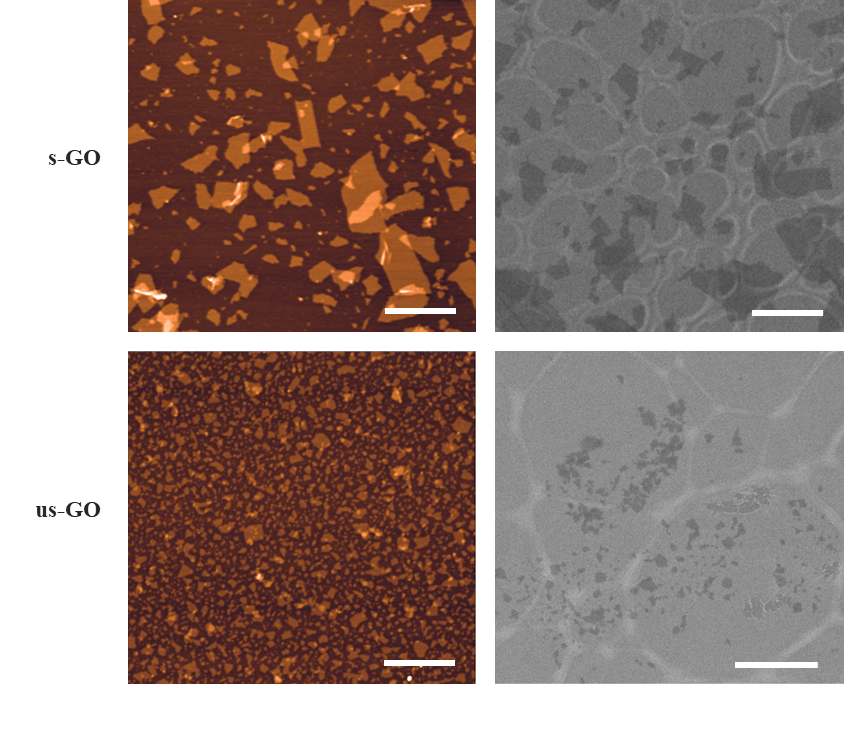


**Figure S1**: Characterisation of GO (s- and us-GO) by atomic force microscopy (left panels) and electron microscopy (right panels). Scale bar = 1 µm. See **Table S1** for the summary of the physico-chemical properties.


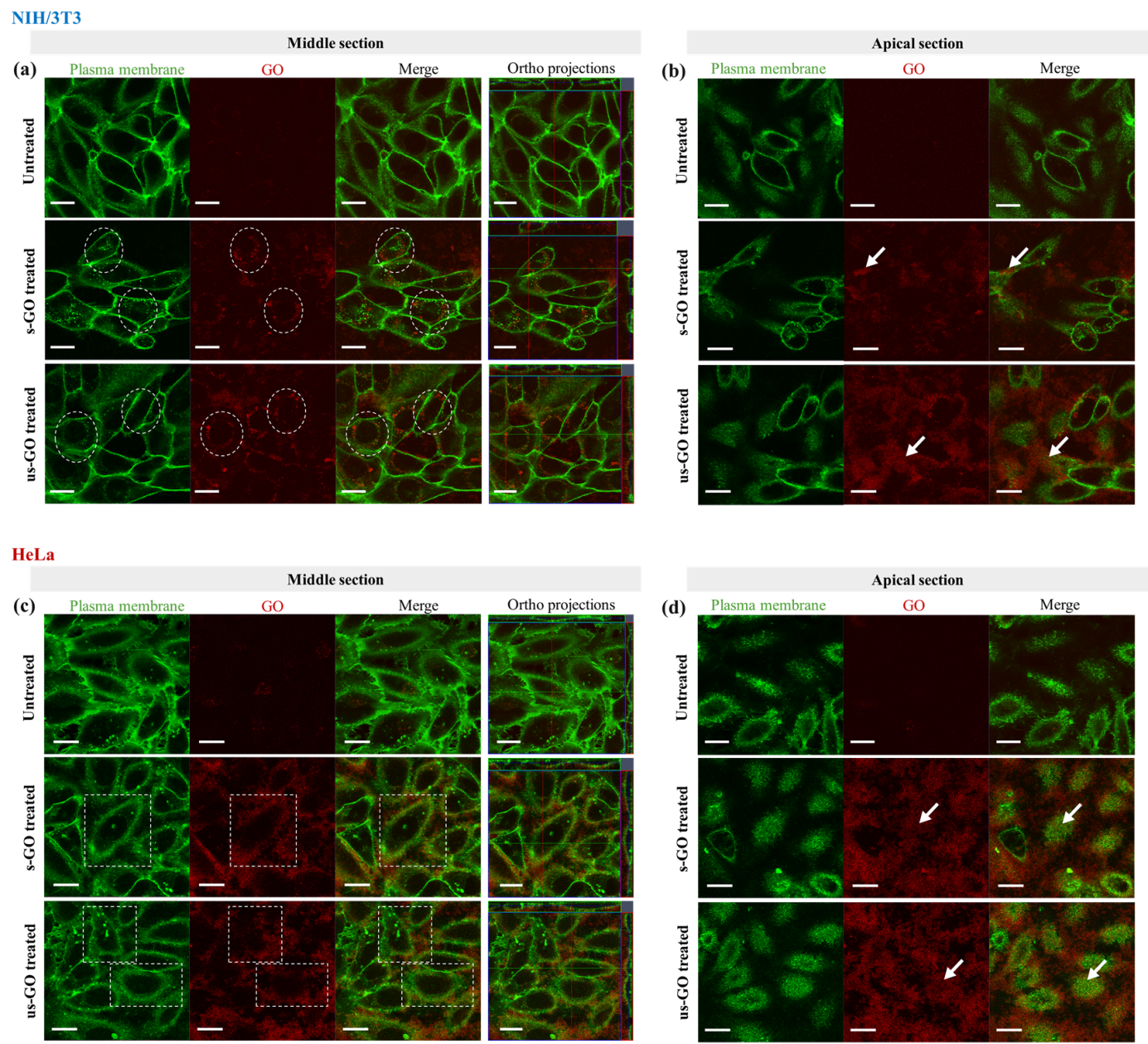


**Figure S2**: Representative images (middle and apical section) of GO (s- and us-GO) interaction profiles in **(a** – **b)** NIH/3T3 and **(c** – **d)** HeLa cells by CLSM. Both s-GO and us-GO (50 μg/mL) were found internalised in NIH/3T3 cells (indicated by white circles) but predominately surrounded the plasma membrane in HeLa cells (indicated by white rectangles). The materials were also found on the plasma membrane (indicated by white arrows) in both cell lines. Green = plasma membrane, red = GO. Scale bar = 20 µm. See **Figures S3** and **S7** for the interaction of s-GO and us-GO at different concentrations with NIH/3T3 and HeLa cells, respectively.

**Table S2**: Summary of GO (s- and us-GO) interaction profile in cancer (HeLa, A549, SW 480, SH-SY5Y, U87 MG, DU-145, PC3 and LNCaP) and non-cancer (BEAS-2B, NIH/3T3, HaCaT, 293T and PNT-2) cells by CLSM.

| **Cell line** | **Tissue/morphology** | **s-GO and us-GO uptake** |
| --- | --- | --- |
| **BEAS-2B** | Normal human lung/epithelial | **+** |
| **NIH/3T3** | Normal mouse embryonic/fibroblasts | **+** |
| **HaCaT** | Normal human skin/epithelial | **+** |
| **293T** | Normal human embryonic kidney/epithelial | **+** |
| **PNT-2** | Normal human prostate/epithelial | **+** |
| **HeLa** | Adenocarcinoma human cervix/epithelial | **–** |
| **A549** | Carcinoma human lung/epithelial | **–** |
| **SW 480** | Adenocarcinoma human cervix/epithelial | **–** |
| **SH-SY5Y** | Neuroblastoma human bone marrow neuroblastoma/epithelial | **–** |
| **U87 MG** | Glioblastoma human brain/epithelial | **–** |
| **DU-145** | Carcinoma human prostate/epithelial | **–** |
| **PC3** | Adenocarcinoma human prostate/epithelial | **–** |
| **LNCaP** | Carcinoma human prostate/epithelial | **–** |


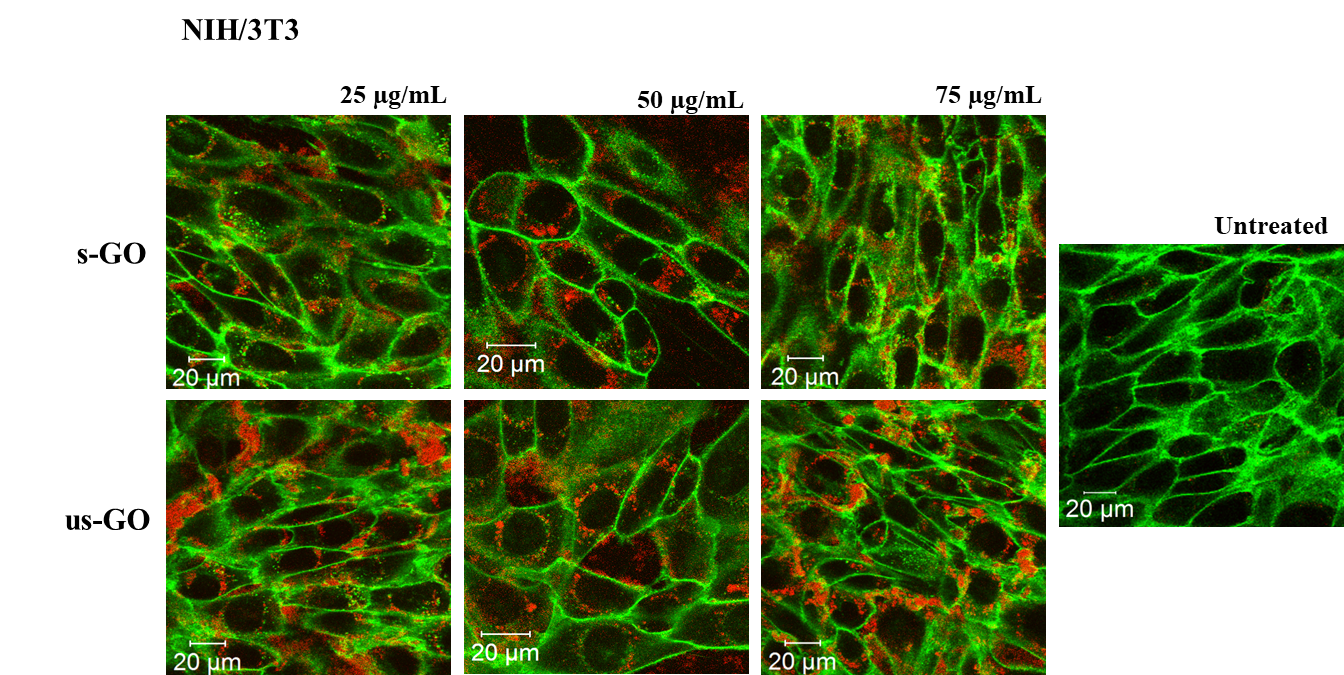


**Figure S3**: Interactions of s-GO and us-GO at 25, 50 and 75 μg/mL with NIH/3T3 cells. Green = plasma membrane, red = GO.


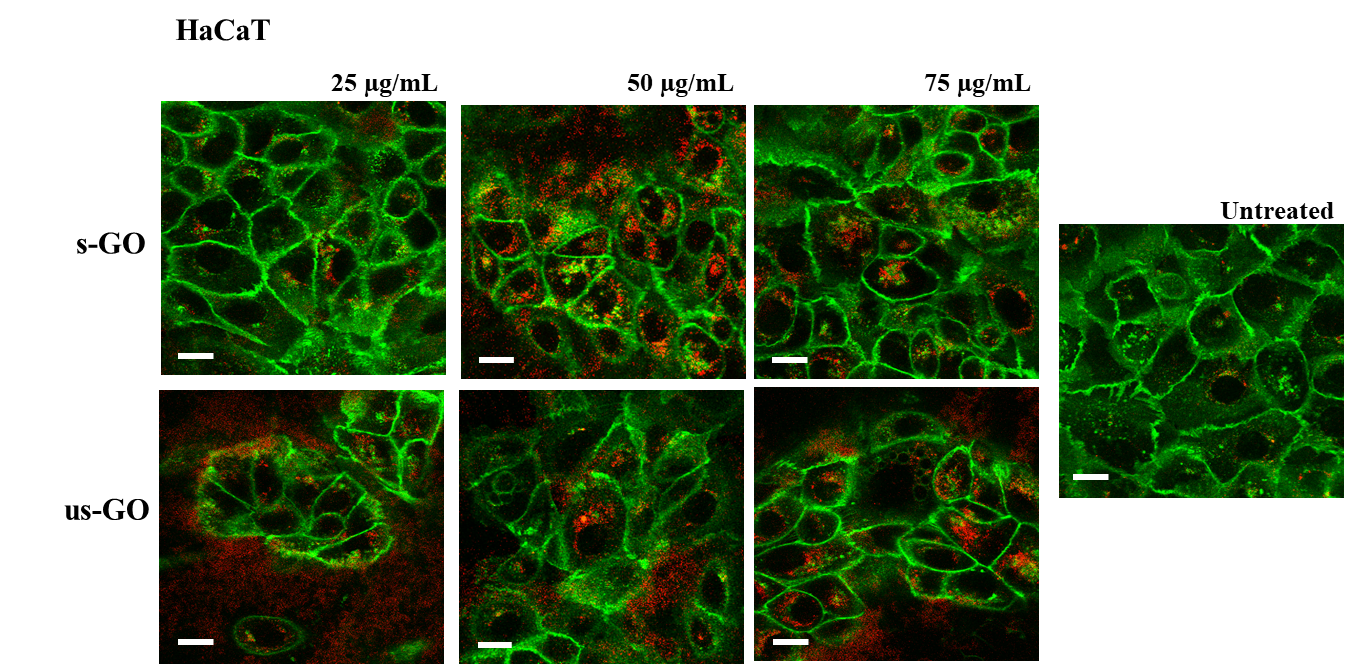


**Figure S4**: Interactions of s-GO and us-GO at 25, 50 and 75 μg/mL with HaCaT cells. Green = plasma membrane, red = GO. Scale bar = 20 μm.


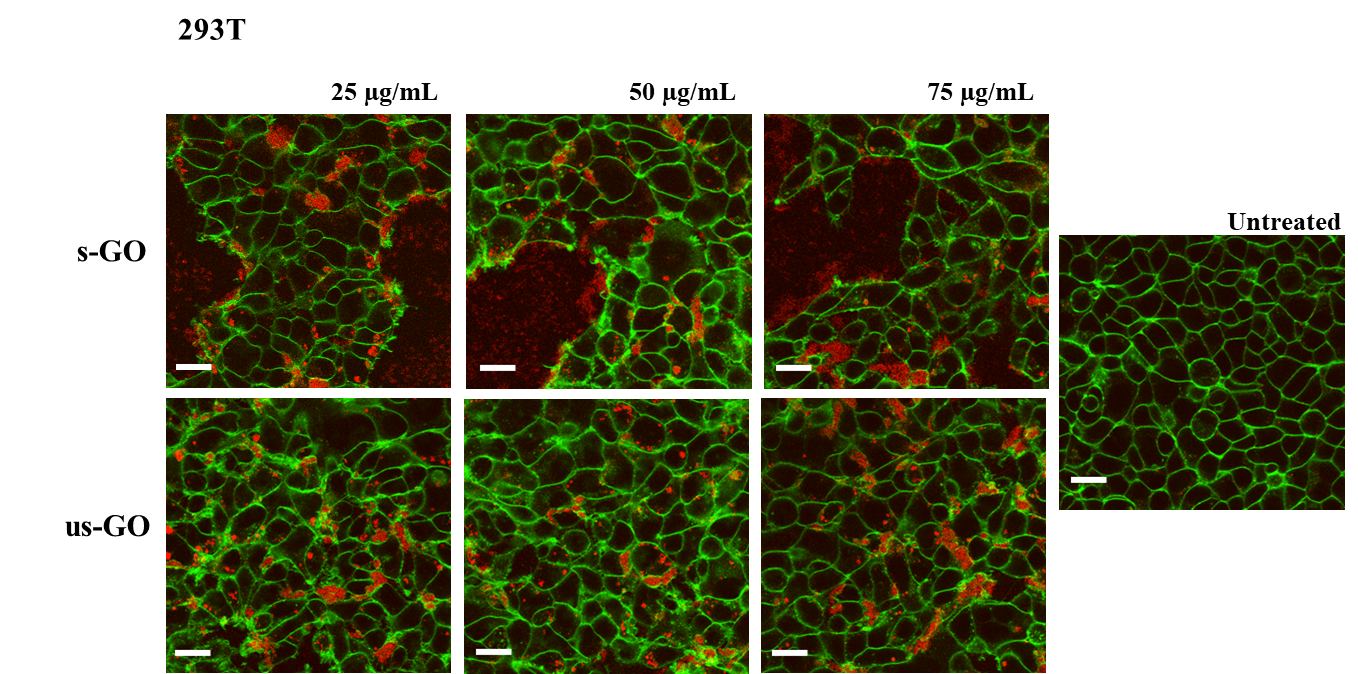


**Figure S5**: Interactions of s-GO and us-GO at 25, 50 and 75 μg/mL with 293T cells. Green = plasma membrane, red = GO. Scale bar = 20 μm.


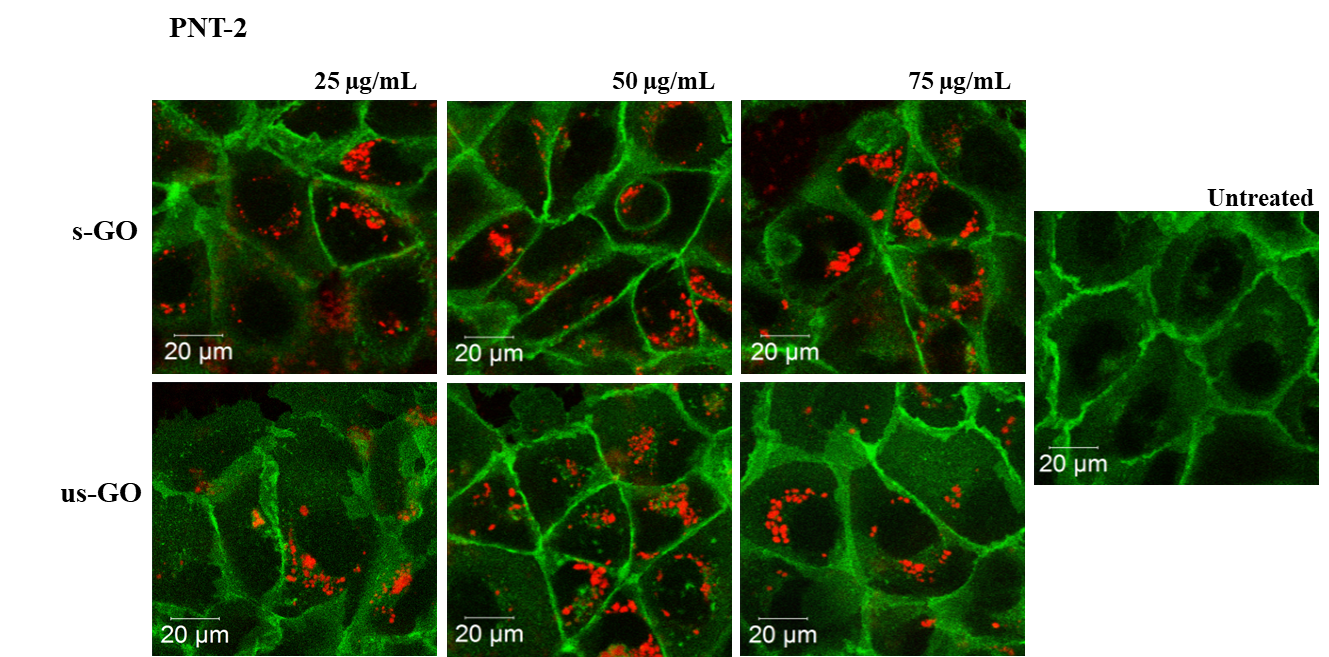


**Figure S6**: Interactions of s-GO and us-GO at 25, 50 and 75 μg/mL with PNT-2 cells. Green = plasma membrane, red = GO.


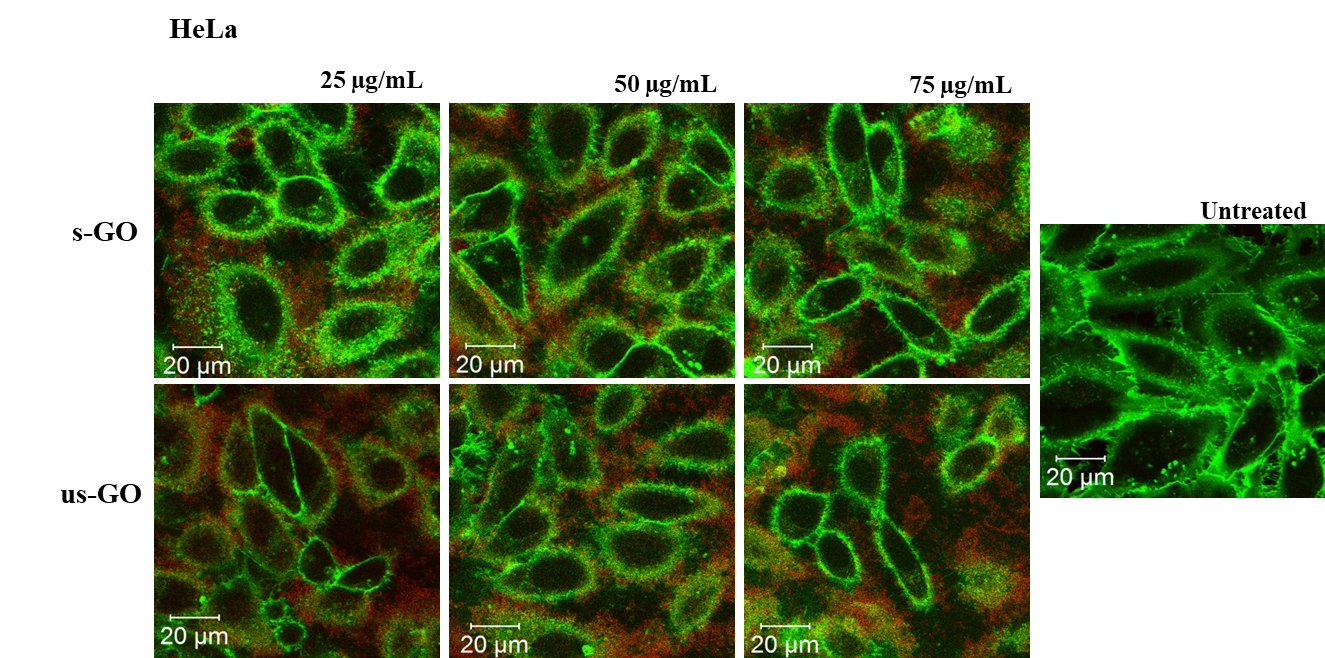


**Figure S7**: Interactions of s-GO and us-GO at 25, 50 and 75 μg/mL with HeLa cells. Green = plasma membrane, red = GO.


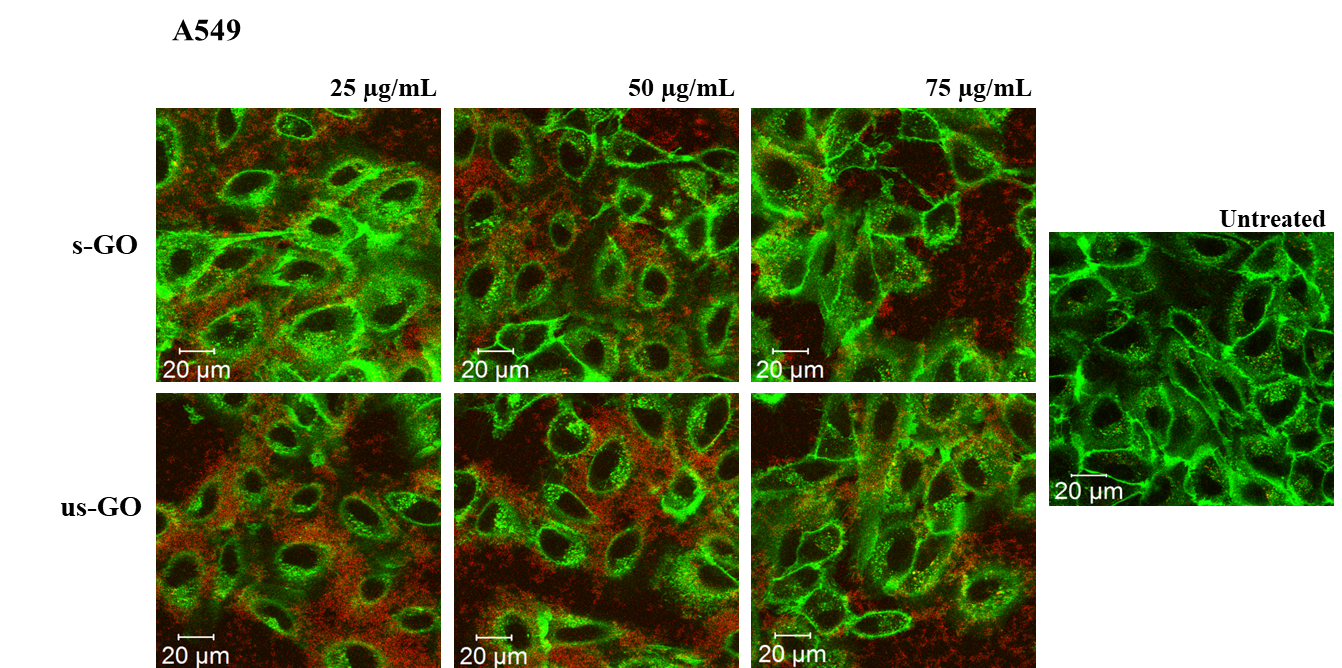


**Figure S8**: Interactions of s-GO and us-GO at 25, 50 and 75 μg/mL with A549 cells. Green = plasma membrane, red = GO.


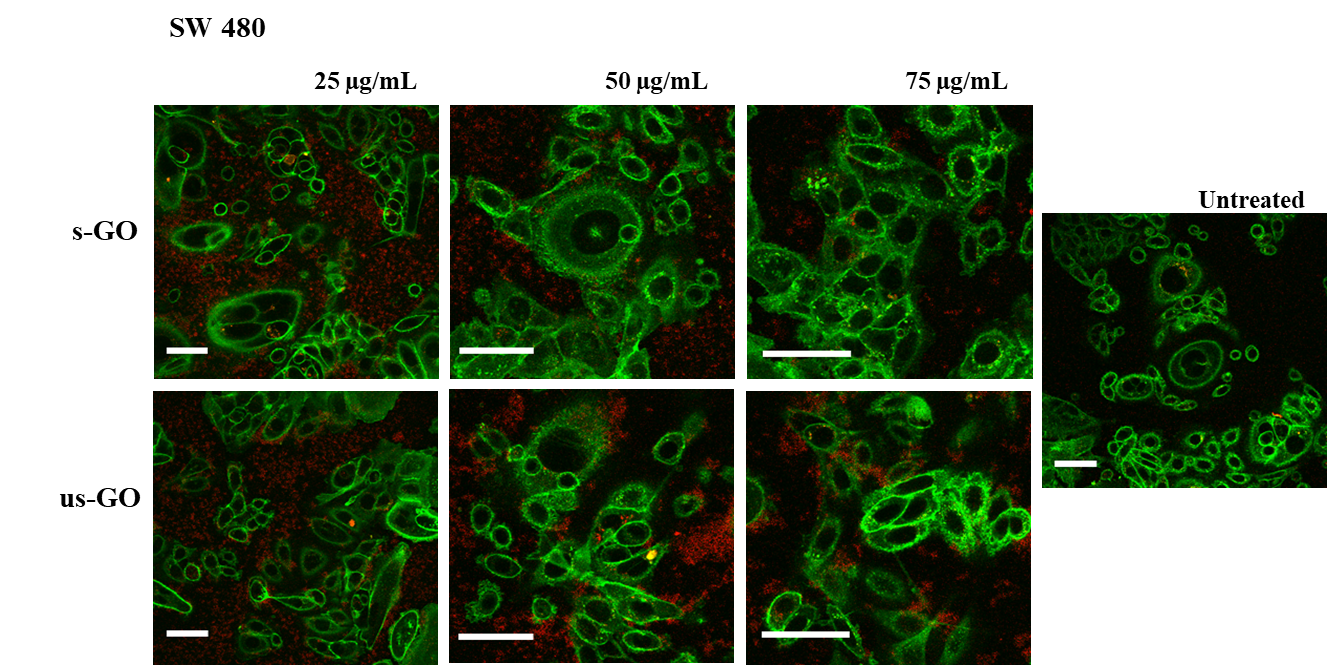


**Figure S9**: Interactions of s-GO and us-GO at 25, 50 and 75 μg/mL with SW 480 cells. Green = plasma membrane, red = GO. Scale bar = 50 μm.


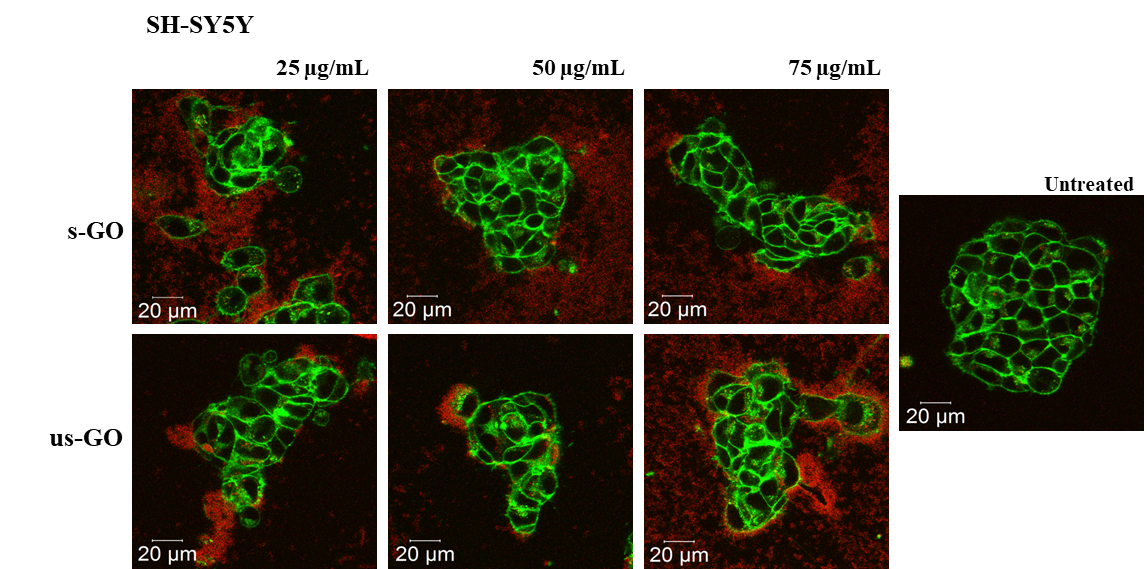


**Figure S10**: Interactions of s-GO and us-GO at 25, 50 and 75 μg/mL with SH-SY5Y cells. Green = plasma membrane, red = GO.


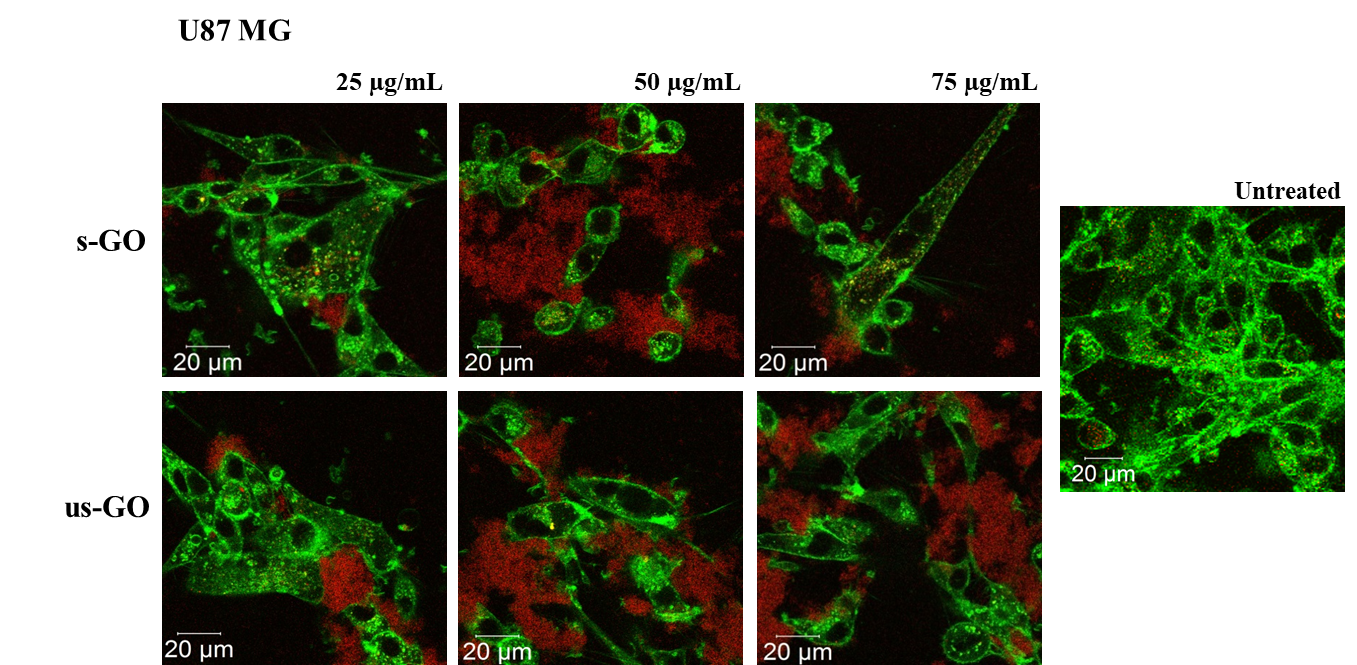


**Figure S11**: Interactions of s-GO and us-GO at 25, 50 and 75 μg/mL with U87 MG cells. Green = plasma membrane, red = GO.


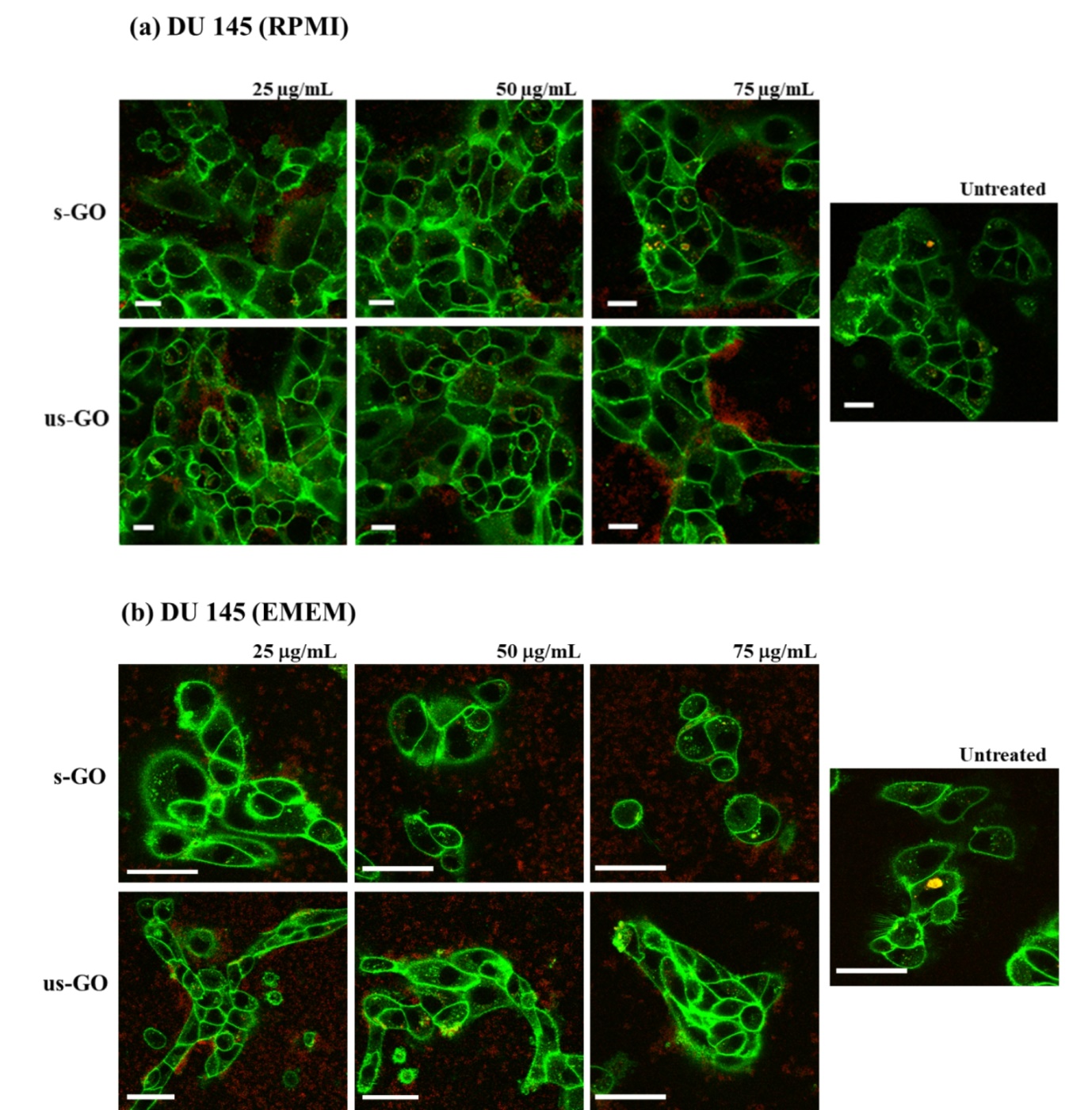


**Figure S12**: Interactions of s-GO and us-GO at 25, 50 and 75 μg/mL with DU 145 cells [in **(a)** RPMI with 10% FBS and **(b)** EMEM with 10% FBS]. Green = plasma membrane, Red = GO. Scale bar = **(a)** 20 μm or **(b)** 50 μm.


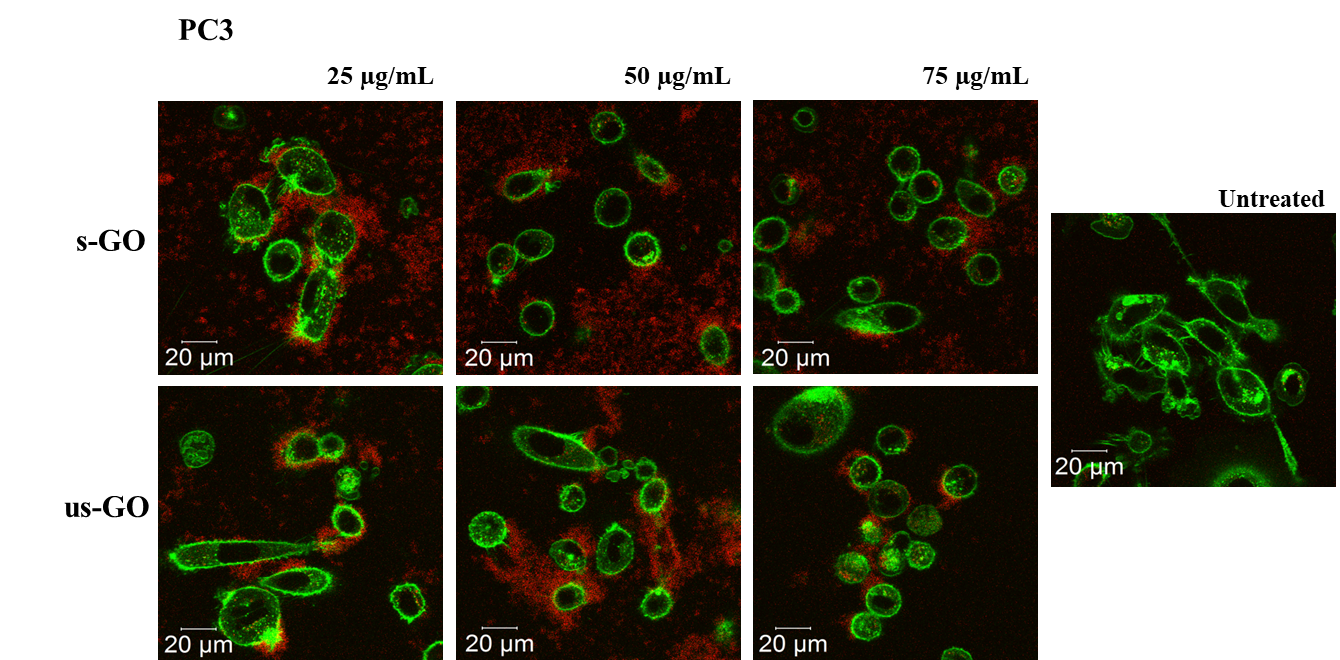


**Figure S13**: Interactions of s-GO and us-GO at 25, 50 and 75 μg/mL with PC3 cells. Green = plasma membrane, red = GO.


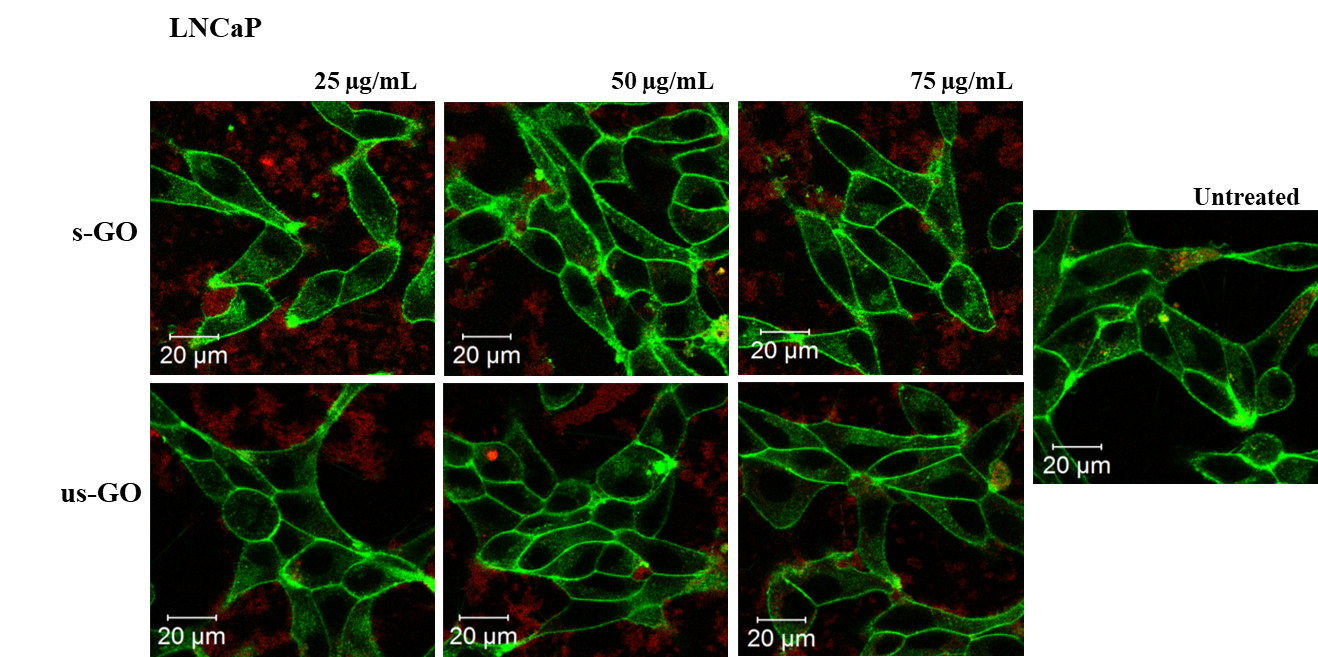


**Figure S14**: Interactions of s-GO and us-GO at 25, 50 and 75 μg/mL with LNCaP cells. Green = plasma membrane, red = GO.


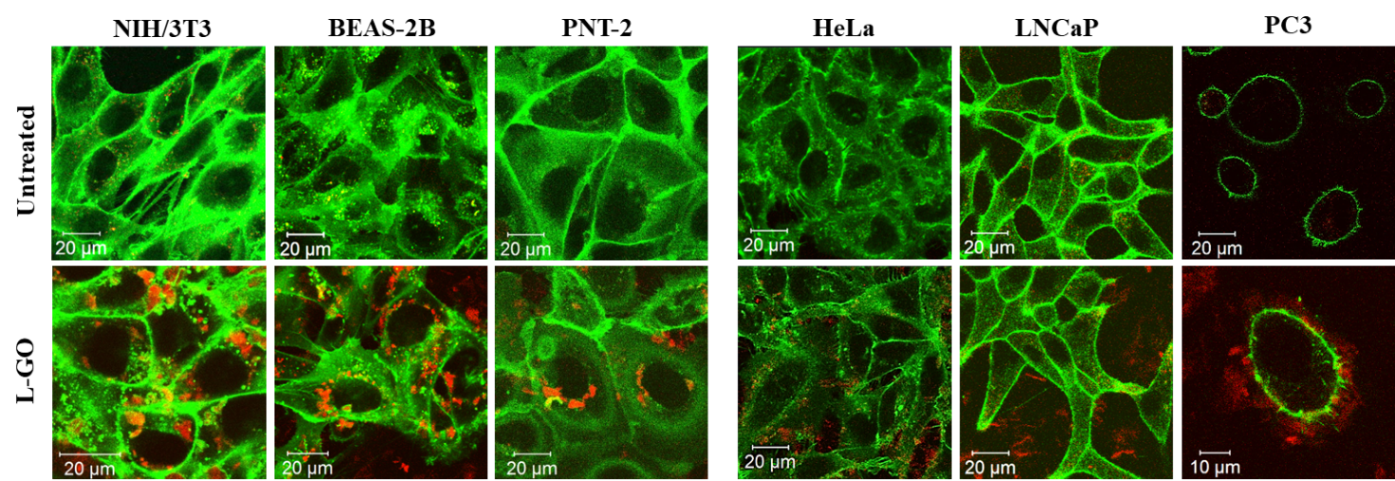


**Figure S15**: Interaction l-GO (50 μg/mL) with non-cancer (NIH/3T3, BEAS2B, PNT-2) and cancer (HeLa, LNCaP and PC3) cell lines by confocal imaging. Green = plasma membrane, red = GO.


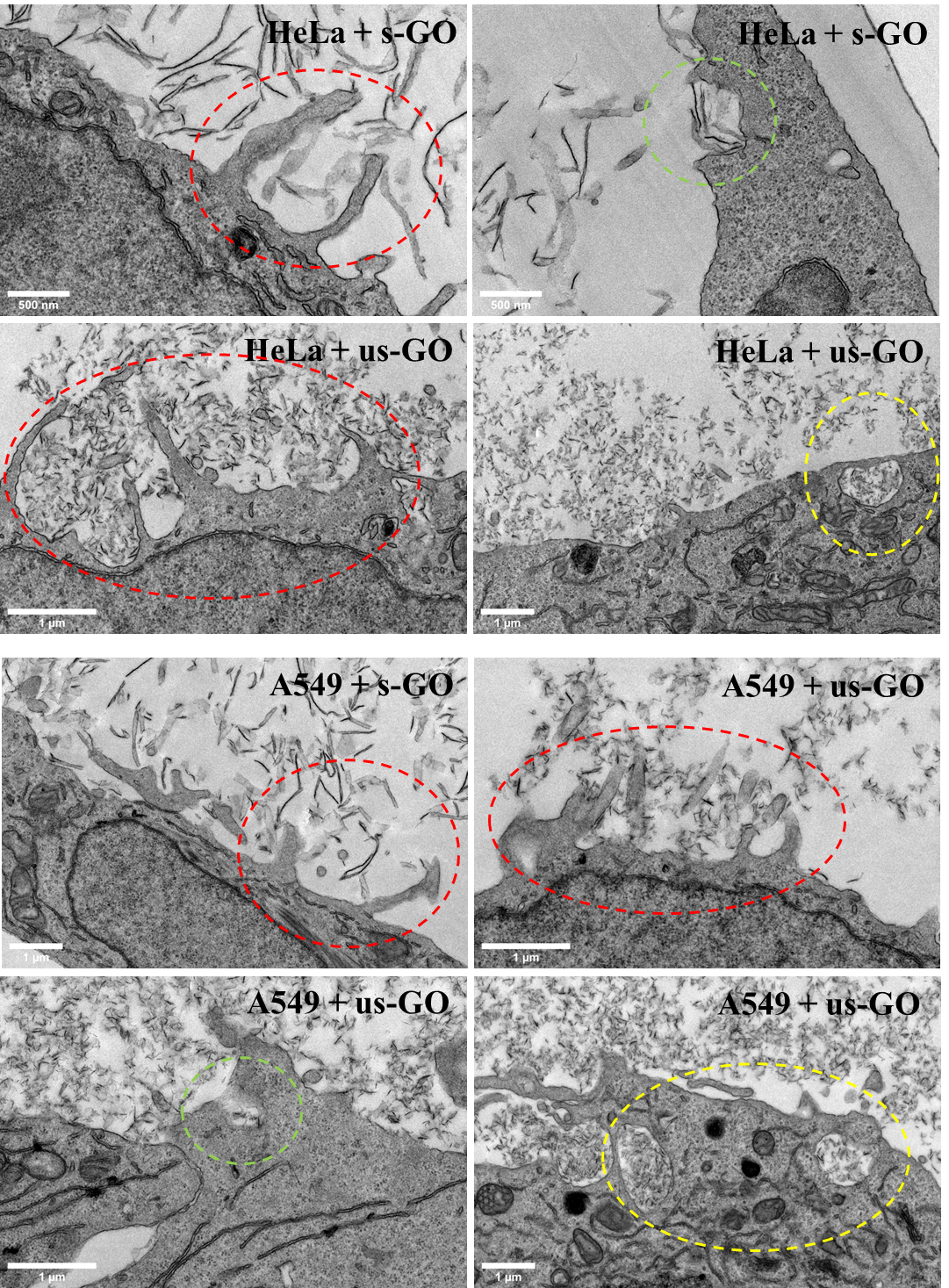


**Figure S16**: Sign of membrane ruffles (indicated by red circles) and membrane invagination (indicated by green circles) was observed in HeLa and A549 cells by TEM imaging. Also, the uptake of us-GO is found in a few cancer cells (indicated by yellow circles).

**Table S3**: Statistical analysis for the interaction of GO (s- and us-GO) with BEAS-2B, NIH/3T3, HeLa and A549 by flow cytometry (cells collected with PBS (-/-) washing); see **Fig. 2e** for the corresponding graph. Tables showed the statistical analysis for the interaction of GO (s- and us-GO): **(a)** compared across the four cell lines, and **(b)** within each cell line. The data were statistically analysed using analysis of variance (two-way ANOVA) with Tukey’s multiple comparisons test. n = 3 with duplicates *Statistically different: NS = non-significant, *****p* < 0.0001.

| **(a)** | s-GO | us-GO |
| --- | --- | --- |
| **HeLa vs A549** | **** | **** |
| **HeLa vs NIH/3T3** |  |  |
| **HeLa vs BEAS-2B** |  |  |
| **A549 vs NIH/3T3** | NS | NS |
| **A549 vs BEAS-2B** |  |  |
| **NIH/3T3 vs BEAS-2B** |  |  |

| **(b)** | **HeLa** | **A549** | **NIH/3T3** | **BEAS-2B** |
| --- | --- | --- | --- | --- |
| s-GO **vs** us-GO | **** | | | |


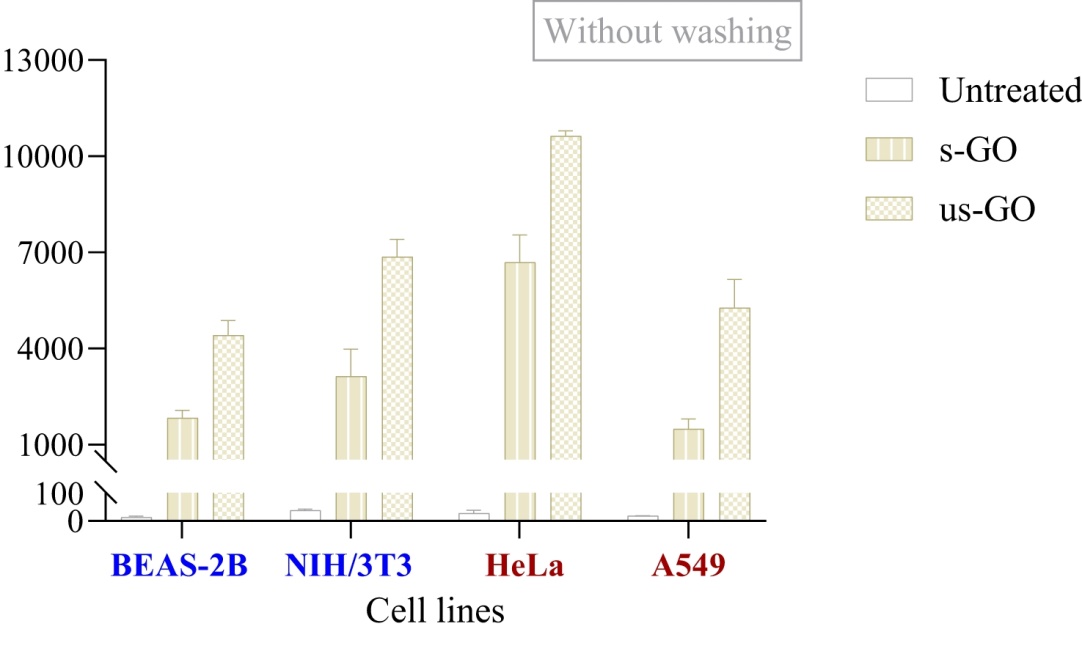


| **(a)** | s-GO | us-GO |
| --- | --- | --- |
| **HeLa vs A549** | **** | **** |
| **HeLa vs NIH/3T3** |  |  |
| **HeLa vs BEAS-2B** |  |  |
| **A549 vs NIH/3T3** | NS | NS |
| **A549 vs BEAS-2B** |  |  |
| **NIH/3T3 vs BEAS-2B** |  |  |

| **(b)** | **HeLa** | **A549** | **NIH/3T3** | **BEAS-2B** |
| --- | --- | --- | --- | --- |
| s-GO **vs** us-GO | **** | | | |

**Figure S17**: Interaction of GO (s- and us-GO) with HeLa, A549, BEAS-2B and NIH/3T3 cells by flow cytometry (cells were collected without PBS (-/-) washing). Tables showed the statistical analysis for the interaction of GO **(a)** compared across the four cell lines and **(b)** within each cell line. The data were statistically analysed using analysis of variance (two-way ANOVA) with Tukey’s multiple comparisons test. n = 3 with duplicates *Statistically different: NS = non-significant, *****p* < 0.0001.

**Figure S18**: Emission spectra of s-GO and us-GO (2 mg/mL) in water (solid lines) and RPMI with 10% FBS (materials freshly prepared in complete medium and re-suspended in water for measurement). Excitation wavelength of 525 nm is used.

**Figure S19**: Emission spectra of the negatively charged (−) 0.1, 0.5, and 1 μm carboxylate-modified, and the positively charged (+) 0.2 and 1 μm amine-modified beads (1.5 uL/mL, in RPMI w 10% FBS). The normalisation factors for each bead type were determined by taking the raw reading at 515 nm, correcting it against the control (RPMI with 10% FBS), and dividing it by the corrected reading for the − 0.1 μm beads (with the lowest intrinsic fluorescent intensity). Subsequently, the raw data from the study of beads-cellular interactions by flow cytometry were divided against the normalisation factors for normalisation (see **Figs. 4** and **S20**). Cells Excitation wavelength of 472 nm was used.


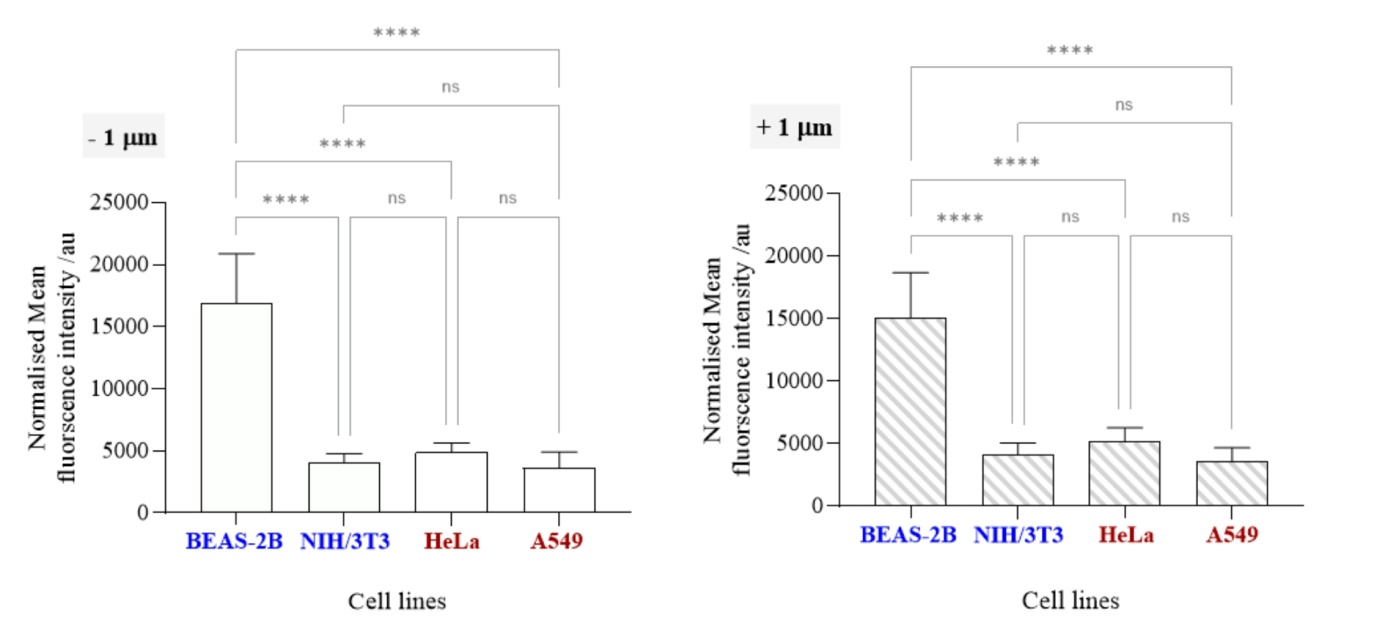


**Figure S20**: Comparison of BEAS-2B, NIH/3T3, HeLa and A549 cells treated with (left) carboxylate-modified or (right) amine-modified 1 μm beads by flow cytometry. The raw data were normalised against the intrinsic fluorescent intensity of the microspheres (see **Figure S19** for the emission spectra of the microspheres). The normalised data were statistically analysed using analysis of variance (one-way ANOVA) with Tukey’s multiple comparisons test. n = 3 with duplicates. *Statistically different: ****p* < 0.001 and *****p* < 0.0001, ns = not significant.


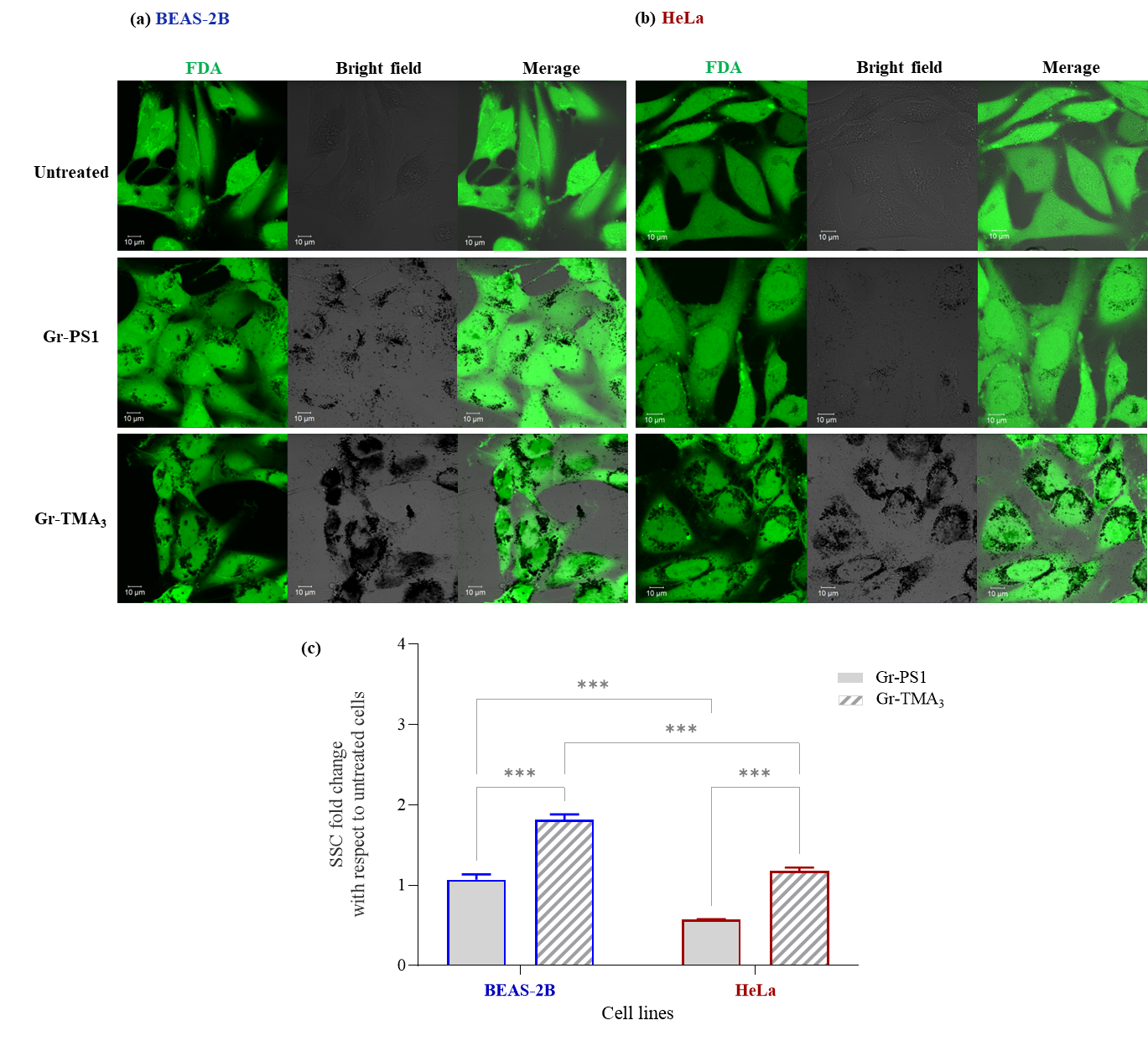


**Figure S21:** Interaction of Gr-PS1 and Gr-TMA_3_ (50 μg/mL, 24 h) in BEAS-2B and HeLa cells by **(a – b)** confocal imaging and **(c)** flow cytometry. Green = FDA dye labelled cells, black = graphene flake. The data were statistically analysed using analysis of variance (two-way ANOVA) with Tukey’s multiple comparisons test. n = 1 with duplicates. *Statistically different: ****p* < 0.001.


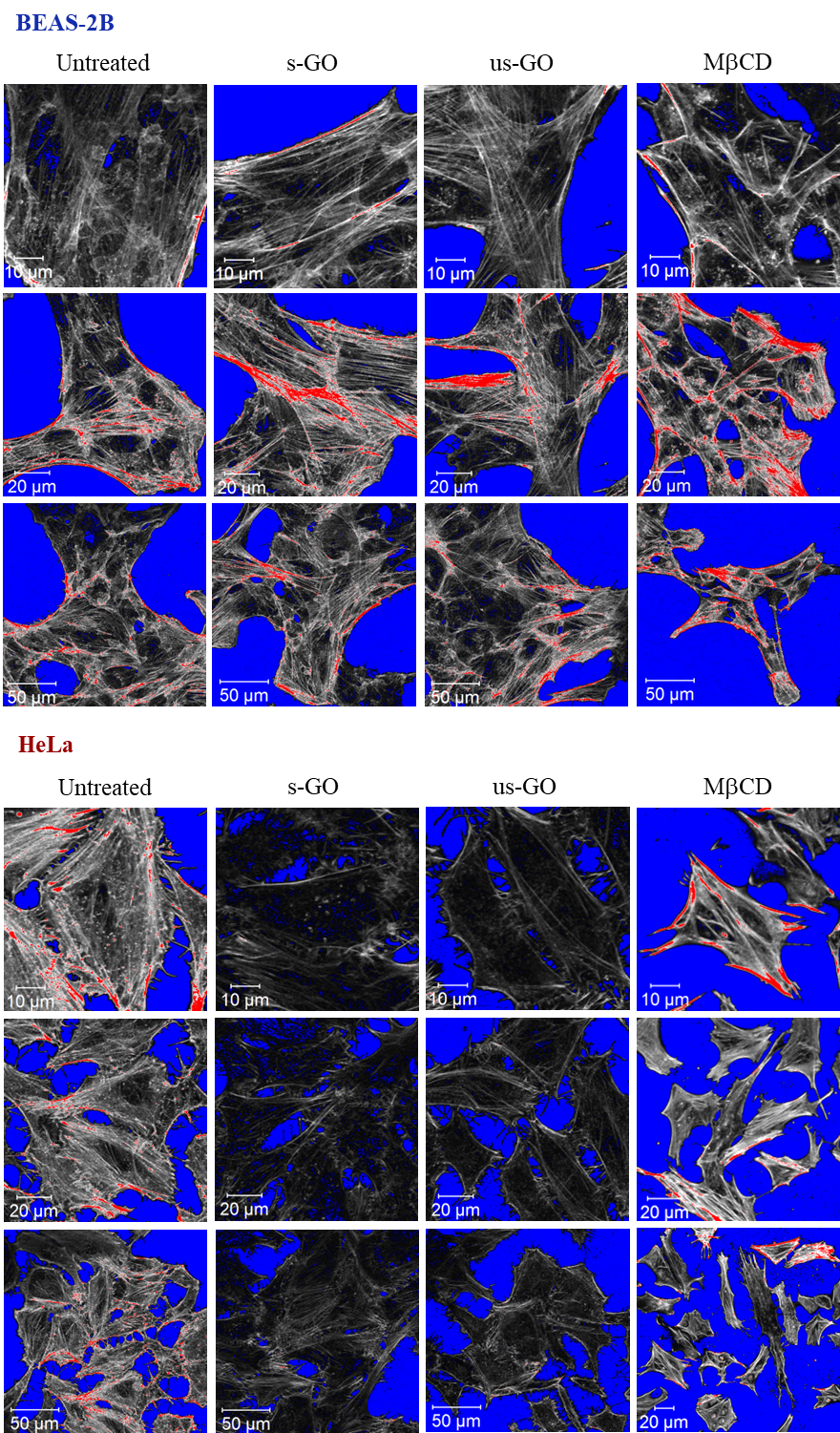


**Figure S22**: Actin filament staining for BEAS-2B and HeLa cells with and without treatment of GO (s-GO or us-GO, 100 μg/mL, 24 h) or M *β*CD (5 mM, 4 h). Images are shown in range indicator (pixels with zero, low, high, and maximum intensity are shown in blue, black, white and red, respectively).


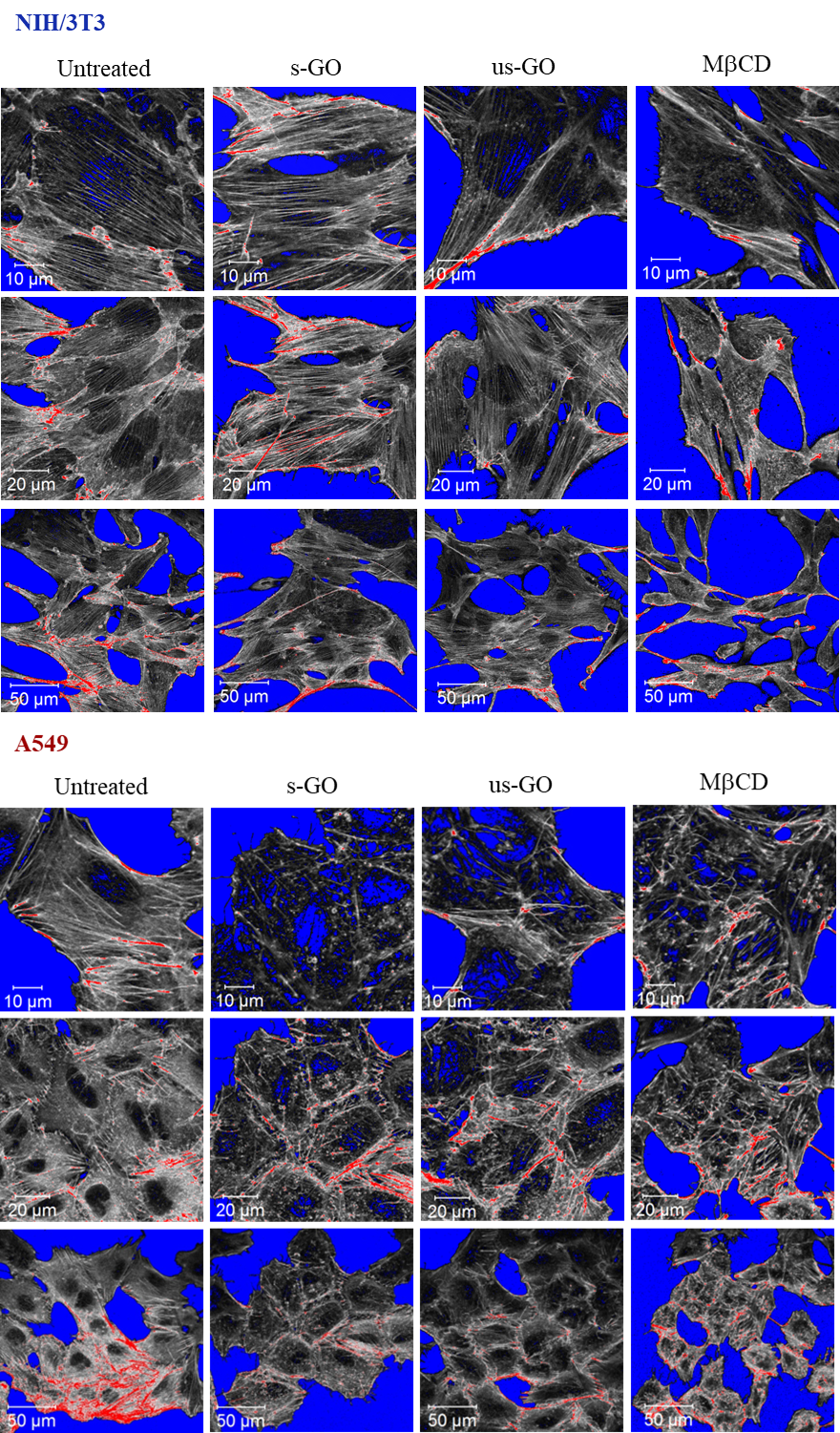


**Figure S23**: Actin filament staining for NIH/3T3 and A549 cells with and without treatment of GO (s-GO or us-GO, 100 μg/mL, 24 h) or M *β*CD (5 mM, 4 h). Images are shown in range indicator (pixels with zero, low, high, and maximum intensity are shown in blue, black, white and red, respectively).
